# Identification and validation of novel biomarkers affecting bladder cancer immunotherapy *via* machine learning and its association with M2 macrophages

**DOI:** 10.3389/fimmu.2022.1051063

**Published:** 2022-11-09

**Authors:** Junkang Wang, Xiaojuan He, Yifeng Bai, Guanghui Du, Minhong Cai

**Affiliations:** ^1^ Department of Outpatient, Sichuan Academy of Medical Sciences and Sichuan Provincial People’s Hospital, Chengdu, Sichuan, China; ^2^ Department of Cancer Center, Sichuan Academy of Medical Sciences and Sichuan People’s Hospital, Chengdu, Sichuan, China; ^3^ Healthcare-associated Infection Management Office, Sichuan Academy of Medical Sciences and Sichuan People’s Hospital, Chengdu, Sichuan, China

**Keywords:** immunotherapy, PTHLH, machine learning, M2 macrophages, bladder cancer

## Abstract

**Background:**

Immunotherapy has shown promising results in bladder cancer therapy options.

**Methods:**

Analysis of open-access data was conducted using the R software. Open-access data were obtained from The Cancer Genome Atlas (TCGA), Gene Expression Omnibus (GEO), and IMvigor210 databases. Immunofluorescence and co-culture systems were utilized to validate the effect of PTHLH on M2 macrophage polarization.

**Results:**

Here, through the combined (TCGA, GSE128959, GSE13507, and GSE83586) and IMvigor210 cohorts, we comprehensively investigated the biological and immune microenvironment differences in patients with diverse immunotherapy responses. Meanwhile, we found that M2 macrophage could affect bladder cancer immunotherapy sensibility. Moreover, based on the machine learning algorithm (LASSO logistics regression), PTHLH, BHMT2, and NGFR were identified, which all have good prediction abilities for patient immunotherapy. Then, a logistics regression model was established based on PTHLH, BHMT2, and NGFR, and each patient was assigned a logistics score. Subsequently, we investigated the difference in patients with high low logistics scores, including biological enrichment, immune microenvironment, and genomic characteristics. Meanwhile, data from the Human Protein Atlas database indicated a higher protein level of PTHLH in bladder cancer tissue. Immunofluorescence indicated that the knockdown of PTHLH in bladder cancer cells can significantly inhibit the M2 polarization of co-culture M0 macrophages.

**Conclusions:**

Our study investigated the difference between bladder cancer immunotherapy responders and non-responders. Meanwhile, the PTHLH was identified as a novel biomarker for bladder cancer immunotherapy.

## Introduction

Bladder cancer is the leading malignancy in the urogenital system and is responsible for a serious health concern globally (1). Bladder cancer usually occurs as a result of several factors such as gender, genetic differences, and lifestyle (1). In bladder cancer, there are two clinical phenotypes: non-muscle-invasive bladder cancer (NIMBC) and muscle-invasive bladder cancer (MIBC). Generally, surgery can provide long-term therapeutic benefits for most NIMBC and high-level MIBC patients. Also, MIBC tends to suffer from worse survival and more limited treatment options compared to NIMBC (2). Clinically, bladder cancer patients often benefit from immunotherapy. Nonetheless, immunotherapy is still ineffective for a substantial number of people, leading to poor outcomes (3).

There is a long history of immunotherapy in bladder cancer treatment. In 1976, researchers found that the intravesical instillation of the BCG vaccine can kill bladder cancer cells by inducing a local immune response (4). However, for decades, bladder cancer management has remained relatively unchanged and the high recurrence rate remains a challenge (5). Simultaneously, the microenvironment in bladder cancer is always immunosuppressive. Bladder cancer often has a high infiltration level of Treg cells and is regulated by multiple cytokines (6). Consequently, studies are underway to identify new targets for immunotherapy for bladder cancer. Shi et al. found that the mutagenesis mediated by APOBEC can effectively indicate the survival and immunotherapy of bladder cancer (7). Groeneveld et al. revealed that CXCL13, a marker of tertiary lymphoid structures, can effectively indicate the survival of advanced bladder cancer patients receiving immunotherapy (8). Yi et al. found that IGFBP7 is associated with immunological characteristics and might be an immunotherapy target for bladder cancer (9). Same as other cancers, bladder cancer uses immune checkpoints to regulate immunity, notably PD-1/L1 and CTLA4. Nowadays, some clinical trials that target immune checkpoints have achieved some promising results in bladder cancer (10). Meanwhile, the combination of chemotherapy and immunotherapy can effectively improve the antitumor effect in bladder cancer (11). Therefore, the exploration of the novel biomarkers involved in bladder cancer immunotherapy is meaningful.

Here, based on the open-access data, we comprehensively investigated the biological and immune microenvironment differences in patients with diverse immunotherapy responses. Meanwhile, we found that M2 macrophage could affect the sensitivity of bladder cancer patients to immunotherapy. Moreover, PTHLH, BHMT2, and NGFR were identified, which all have good prediction abilities on patient immunotherapy. Then, a logistics regression model was established based on PTHLH, BHMT2, and NGFR. Next, we explored the difference between patients with high and low logistics scores, including biological enrichment, immune microenvironment, and genomic characteristics. Immunofluorescence showed that the knockdown of PTHLH in bladder cancer cells can significantly inhibit the M2 polarization of co-culture M0 macrophages, making it a potential biomarker for bladder cancer.

## Methods

### Data collection

Open-access data were obtained from The Cancer Genome Atlas (TCGA), Gene Expression Omnibus (GEO), and IMvigor210 databases. As for the patients in TCGA, the expression profile was in “STAR-Counts” form and clinical data were in “bcr-xml” form. Before analysis, all the data were pre-processed. For the dataset in GEO, the criteria “1. Sample counts > 150; 2. Complete expression profile; 3. Complete survival information” were used for data filtering. Ultimately, GSE128959, GSE13507, and GSE83586 were identified and annotated according to their platforms. The sva package was utilized for data combination. Levels of tumor mutational burden (TMB) and microsatellite instability (MSI) were extracted from the genomic data of the TCGA database. The IMvigor210 dataset was downloaded from http://research-pub.gene.com/IMvigor210CoreBiologies. Baseline information of the included samples is shown in **Supplementary Tables S1**-**S4**.

### Immunotherapy evaluation

Response of patients on immunotherapy was conducted using the Tumor Immune Dysfunction and Exclusion (TIDE) analysis (12). The input file was the expression profile, and according to this, each patient owns a TIDE score. The immunotherapy responders were those whose TIDE score > 0; otherwise, non-responders.

### Machine learning algorithm

The machine learning algorithm, LASSO logistic regression, was used for optimal variable selection (13). For the identified characteristic genes, the glm function in R software was used for logistics model construction. The “family” was set as “binomial”.

### Biological enrichment

Biological enrichment difference was identified using the Gene Set Enrichment Analysis (GSEA), whose gene sets for reference were “Hallmark” and “c2.cp.kegg.v2022.1.Hs.symbols” (14). Implementation of gene ontology (GO) analysis was based on the clusterprofiler package (15). The terms of adjusted *p*-value < 0.05 was considered statistically significant.

### Immune microenvironment exploration

CIBERSORT was utilized to quantify the infiltration level of 22 immune cells based on the input transcriptional profile data (16). Calculation of the immune score and the stromal score was conducted using the Estimate packages in R software.

### Genomic difference

The index reflecting tumor stemness characteristics named mRNAsi was extracted from the previous study (17). The frequency and somatic copy number alteration (SCNA) level difference of human chromosomes was extracted from the TCGA database.

### Immunohistochemistry

Evaluation of protein levels of PTHLH, BHMT2, and NGFR was conducted using representative immunohistochemistry images (normal bladder and bladder cancer sections) obtained from the Human Protein Atlas (HPA) database.

### Cell culture and transfection

Cell lines T24 and THP-1 were laboratory stock and cultured under routine culture conditions. Lipofectamine 2000 was selected for cell transfection following the standard steps. The following were the sequences of shRNAs used: shRNA1: 5’-CCCTGATTGTGCCATAAAT-3’; shRNA2: 5’-GGCCAGAACAATGAAGAAA-3’; shRNA3: 5’-GGCACTTAGAAGAACCAAT-3’.

### Co-culture system and THP-1 differentiation

THP-1 cells were seeded into six-well plates for 24 h (100 ng/ml PMA) to differentiate into adherent M0 macrophages. The transfected T24 cell lines and differentiated M0 macrophages together constituted a co-culture system. The culture supernatant of transfected T24 cells was collected and added to the M0 macrophages. After that, for 48 h, macrophages were collected for further assay.

### Immunofluorescence

The collected M0 macrophages were firstly cultured on a glass slide and then fixed with 4% PFA at room temperature. Afterwards, 0.05% Triton X-100 was used for cell permeabilization (2 min) and 5% BSA was used for cell blocking (1 h). Next, cells were incubated with the following specific antibodies overnight at 4°C: anti-F4/80 antibody (1:200) and anti-CD206 (1:200) antibody. DAPI was utilized for cell nuclear staining. Fluorescence microscopy was used to visualize immunofluorescence.

### Statistical analysis

Calculation of statistical significance in the different analysis was conducted using the R and GraphPad Prism software. The threshold of statistical significance was 0.05. According to the data distribution, Student’s *t*-test and Mann–Whitney *U* test were tested.

## Results

### Data preparation

**Figure 1:**
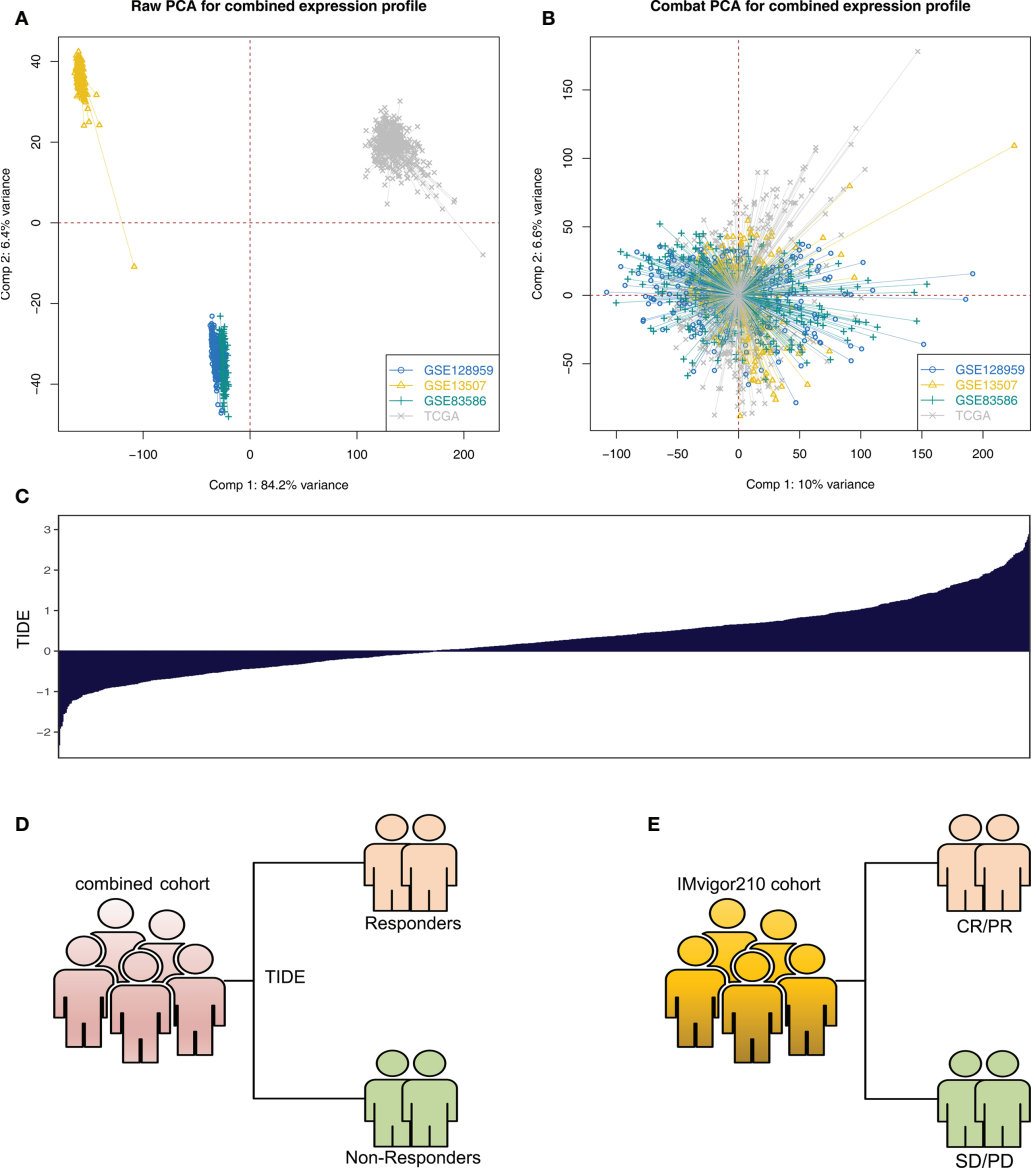
Data preparation **(A)** Significant batch effect of four independent bladder cancer cohorts was observed, namely, TCGA, GSE128959, GSE13507, and GSE83586. **(B)** Sva package was used for data combination and removal of batch difference. **(C)** Overview of TIDE score in the combined cohort (TCGA + GSE128959 + GSE13507 + GSE83586). **(D)** According to TIDE algorithm, the patients in the combined cohort were divided into immunotherapy responders and non-responders. **(E)** SD/PD and CR/PR patients in the IMvigor210 cohort.

The whole flowchart is shown in **Figure S1**. Firstly, the independent bladder cancer cohorts, TCGA, GSE128959, GSE13507, and GSE83586, were selected for our analysis, which showed a high batch effect (**Figure 1A**; Comp 1: 84.2% variance, Comp 2: 6.4% variance). Through the sva package, we effectively decreased the batch difference and completed data combination (**Figure 1B**; Comp 1: 10% variance, Comp 2: 6.6% variance). Furthermore, TIDE analysis was utilized to assess the immunotherapy response (**Figure 1C**). In the combined cohort, the TIDE score was utilized to divide patients into immunotherapy responder and non-responder groups (**Figure 1D**). Meanwhile, in the real immunotherapy IMvigor210 cohort, the bladder patients with SD (stable disease)/PD (progressive disease) and CR (partial response)/PR (complete response) to immunotherapy were enrolled in our analysis (**Figure 1E**).

### Biological difference between the immunotherapy responders and non-responders

**Figure 2:**
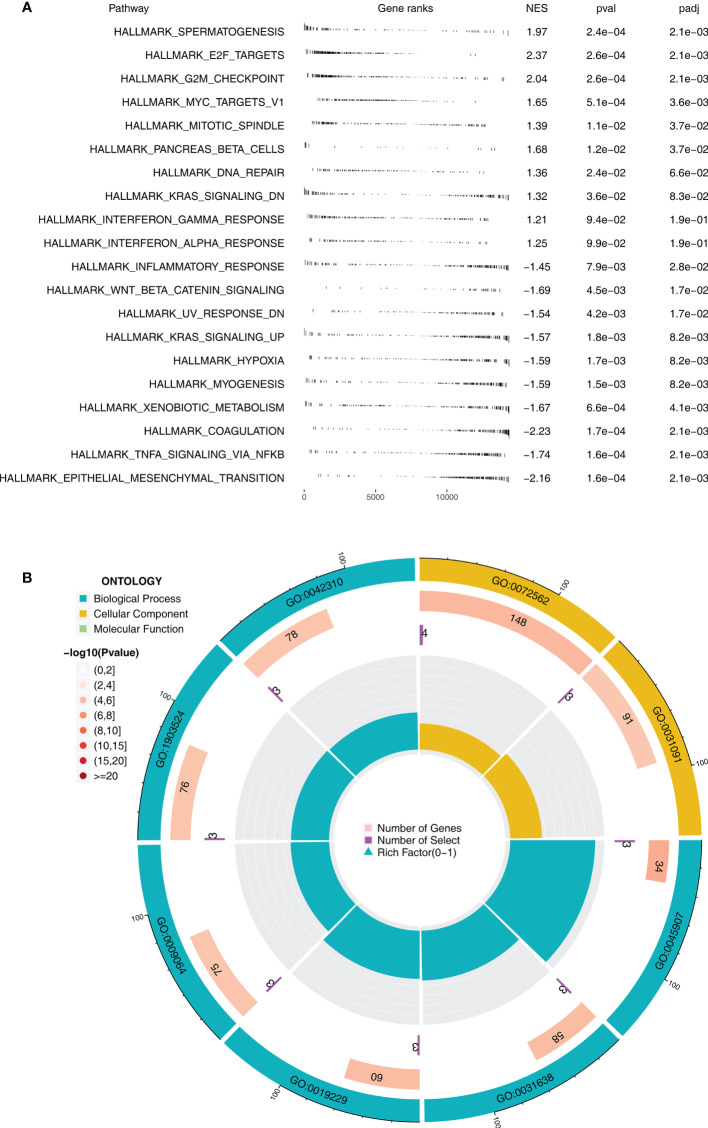
Biological enrichment between immunotherapy responders and non-responders **(A)** GSEA between immunotherapy responders and non-responders based on the Hallmark gene set. **(B)** GO analysis using the clusterprofiler package.

GSEA indicated that, in the immunotherapy responders, pathways of spermatogenesis, E2F target, G2/M checkpoint, MYC target, and mitotic spindle were significantly enriched (**Figure 2A**). Moreover, results of GO analysis revealed that the terms blood microparticle (GO:0076562), positive regulation of vasoconstriction (GO:0045907), zymogen activation (GO:0031638), regulation of vasoconstriction (GO:0019229), positive regulation of blood circulation (GO:1903524), and vasoconstriction (GO:0042310) were remarkably enriched in the immunotherapy responders (**Figure 2B**). Kyoto Encyclopedia of Genes and Genomes (KEGG) analysis indicated that in the immunotherapy responders, the terms antigen processing and presentation, cell cycle, olfactory transduction, DNA replication, natural killer cell-mediated cytotoxicity, and spliceosome were significantly enriched (**Figure S2**).

### M2 macrophages is associated with immunotherapy response

**Figure 3:**
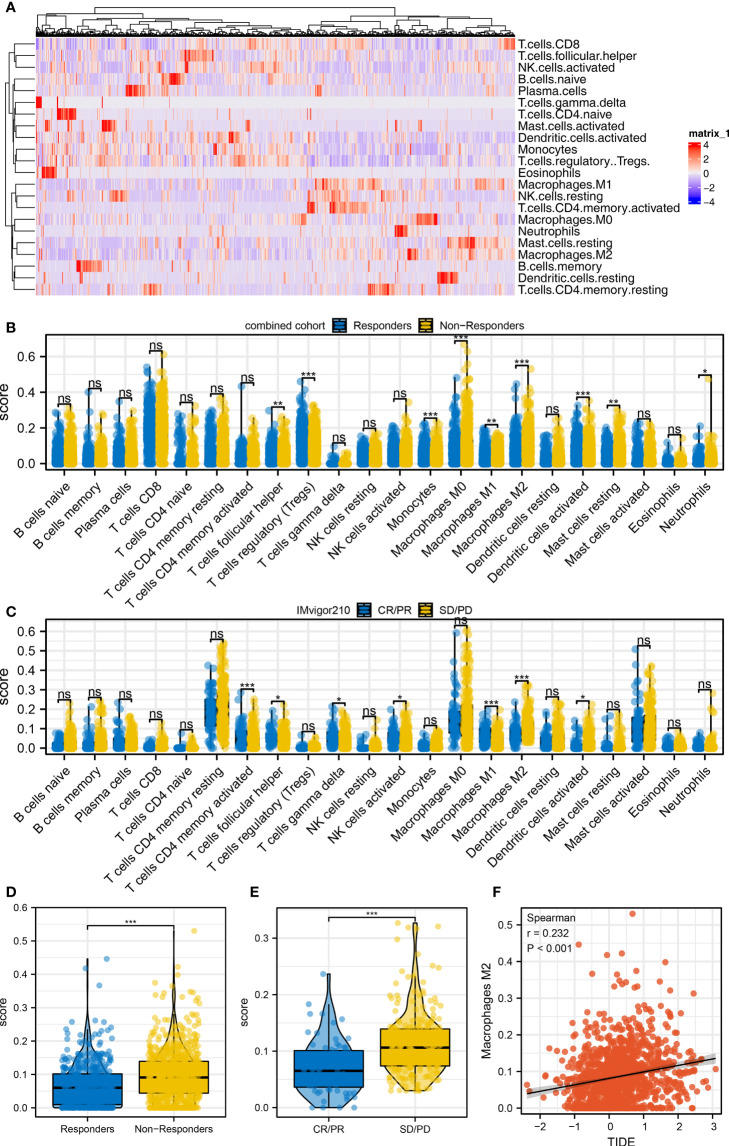
Immune microenvironment difference between immunotherapy responders and non-responders **(A)** The CIBERSORT algorithm was utilized to quantify the immune microenvironment of bladder cancer tissue. **(B)** The level of quantified immune cells in the immunotherapy responders and non-responders (combined cohort), ns = *p* > 0.05, *p < 0.05, **p < 0.01, ***p < 0.001. **(C)** The level of quantified immune cells in the immunotherapy CR/PR and SD/PD patients (IMvigor210 cohort), ns = *p* > 0.05, *p < 0.05, **p < 0.01, ***p < 0.001. **(D)** The level of M2 macrophage in immunotherapy responders and non-responders, ***p < 0.001. **(E)** The level of M2 macrophage in immunotherapy SD/PD and CR/PR patients, ***p < 0.001. **(F)** Correlation between the M2 macrophage and TIDE score.

Immune cell infiltration was quantified using the CIBERSORT algorithm, which is shown in **Figure 3A**. Results indicated that in the combined cohort, immunotherapy non-responders might have a higher level of follicular helper T cells, monocytes, M2 macrophages, activated dendritic cells, and neutrophils, but a lower level of Tregs (**Figure 3B**). Also, in the IMvigor210 cohort, the SD/PD patients might have higher activated memory CD4 T cells, follicular helper T cells, delta gamma T cells, activated NK cells, M2 macrophage, and activated dendritic cells (**Figure 3C**). In the combined and IMvigor210 cohorts, M2 macrophages showed a consistent trend (**Figures 3D, E**). Correlation analysis also indicated a positive correlation between M2 macrophages and TIDE score (**Figure 3F**, *R* = 0.232). The results showed that M2 macrophages might hamper the sensitivity of bladder cancer immunotherapy.

### Identification of the important molecules involved in bladder cancer immunotherapy

**Figure 4:**
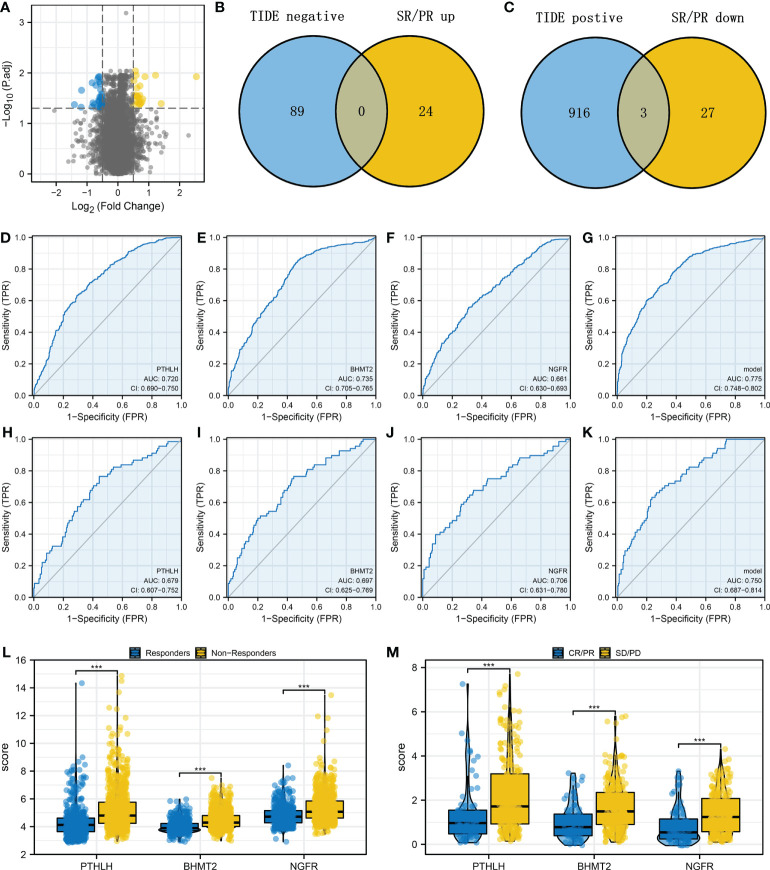
Identification of the PTHLH, BHMT2, and NGFR **(A)** Limma package was used for DEG analysis between the SD/PD and CR/PR patients with the threshold of |logFC| > 0.5 and adjusted *p*-value < 0.05. **(B)** Intersection of the genes negatively correlated with TIDE score and upregulated in CR/PR (LASSO logistics regression). **(C)** Intersection of the genes positively correlated with TIDE score and downregulated in CR/PR (LASSO logistics regression). **(D–G)** Performance of PTHLH, BHMT2, NGFR, and the logistics model in predicting bladder cancer immunotherapy (combined cohort). **(H–K)** ROC curves were used to evaluate the performance of PTHLH, BHMT2, NGFR, and the logistics model in predicting bladder cancer immunotherapy (IMvigor210 cohort). **(L)** The level of PTHLH, BHMT2, and NGFR in immunotherapy responders and non-responders. **(M)** The level of PTHLH, BHMT2, and NGFR in immunotherapy SD/PD and CR/PR patients. ***P < 0.001.

Next, we identified differentially expressed gene (DEG) analysis between SD/PD and CR/PR in the IMvigor210 cohort. Under the threshold of |logFC| > 0.5 and adjusted *p*-value < 0.05, 30 downregulated and 24 upregulated genes were determined (**Figure 4A**). Additionally, we identified that the genes remarkably correlated with TIDE score in the combined cohort (**Table S1**). Then, we intersected the molecules meeting the criteria of “Positive with TIDE and downregulated in CR/PR group” and “Negative with TIDE and upregulated in CR/PR group” based on the machine learning algorithm (**Figures 4B, C**, and **Figure S3**). Finally, three genes were identified, including PTHLH, BHMT2, and NGFR. Receiver operating characteristic (ROC) curves indicate a good prediction ability of these three molecules on patients’ immunotherapy. In the combined cohort, the AUCs of PTHLH, BHMT2, and NGFR were 0.720, 0.735, and 0.661, respectively (**Figures 4D–F**). A logistic regression model was constructed with the formula “−4.9842 + 0.3738*PTHLH + 0.675*BHMT2 + 0.1128*NGFR”, which showed satisfactory prediction efficiency (**Figure 4G**, AUC = 0.775). Also, in the IMvigor210 cohort, the AUCs of PTHLH, BHMT2, NGFR, and logistics scores were 0.679, 0.697, 0.706, and 0.750, respectively (**Figures 4H–K**). A higher level of PTHLH, BHMT2, and NGFR was observed in immunotherapy non-responders (combined cohort) and SD/PD patients (IMvigor210 cohort) (**Figures 4L, M**).

### The logistics score affects patients’ immunotherapy and prognosis

**Figure 5:**
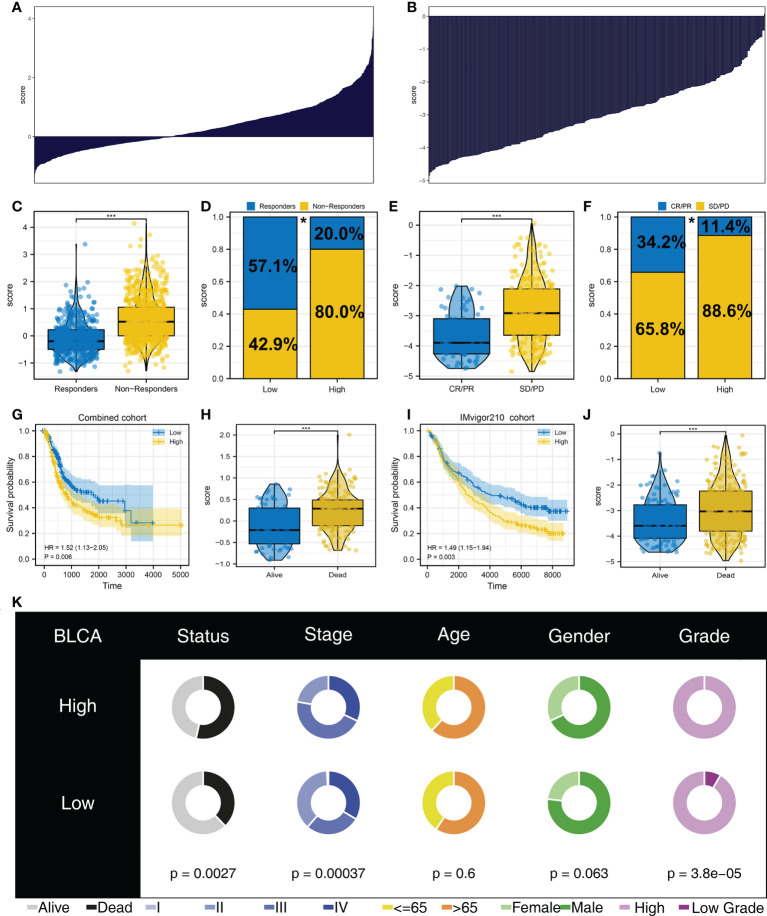
Logistics score was associated with patient immunotherapy response and prognosis **(A)** Calculated logistics score in the combined cohort. **(B)** Calculated logistics score in the IMvigor210 cohort. **(C)** Logistics score in immunotherapy responders and non-responders (combined cohort). **(D)** Percentage of immunotherapy responders and non-responders in patients with a high and those with a low logistics score (combined cohort), *p < 0.05. **(E)** Logistics score in immunotherapy SD/PD and CR/PR patients (IMvigor210 cohort). **(F)** Percentage of immunotherapy SD/PD and CR/PR in patients with a high and those with a low logistics score (IMvigor210 cohort), *p < 0.05. **(G)** Kaplan–Meier survival curves in patients with a high and those with a low logistics score (combined cohort). **(H)** The level of logistics score in alive and dead cases in the combined cohort. **(I)** Kaplan–Meier survival curves in patients with a high and those with a low logistics score (IMvigor210 cohort). **(J)** The level of logistics score in alive and dead cases in the IMvigor210 cohort. **(K)** Clinical correlation analysis of logistics score. ***P < 0.001.

The overview of logistics calculated with the above formula is shown in **Figures 5A, B**. In the combined cohort, patients in the immunotherapy non-responder group had a higher logistics score (**Figure 5C**). Moreover, in patients with a high logistics score, a higher proportion of immunotherapy non-responders was found (**Figure 5D**, 88.6% *vs*. 65.8%). Meanwhile, in the IMvigor210 cohort, we observed the same trend in SD/PD patients (**Figures 5E, F**, 88.6% *vs*. 65.8%). Kaplan–Meier survival curve indicated that the logistics score was correlated with worse survival in both combined and IMvigor210 cohorts and dead cases might have a higher logistics score (**Figures 5G–J**). Clinical correlation analysis revealed that the logistics score was associated with a more progressive clinical stage and grade (**Figure 5K**).

### Immune microenvironment difference in patients with a high and those with a low logistics score

**Figure 6:**
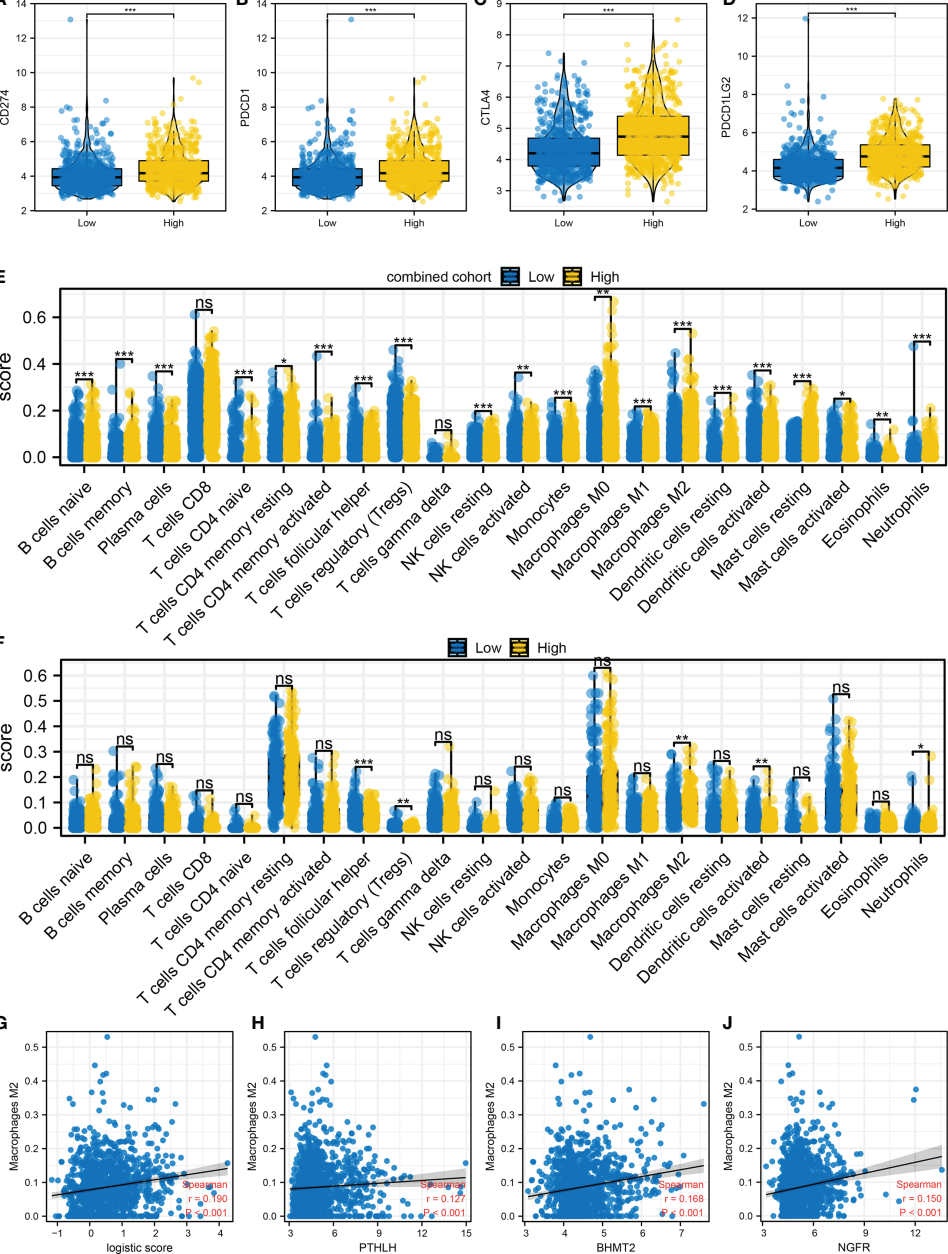
Immune microenvironment difference in patients with a high and those with a low logistics score **(A–D)** Hub immune checkpoints in patients with a high and those with a low logistics score. **(E)** The level of quantified immune cells in patients with a high and those with a low logistics score (combined cohort). **(F)** The level of quantified immune cells in patients with a high and those with a low logistics score (IMvigor210 cohort). **(G)** Correlation between logistics score and M2 macrophages. **(H–J)** Correlation between PTHLH, BHMT2, NGFR, and M2 macrophages. *P < 0.05, **P < 0.01, ***P < 0.001. ns: P > 0.05.

Immune checkpoint is tightly correlated with the immunotherapy response. Therefore, we evaluated the level of hub immune checkpoints (CD274, PDCD1, CTLA, and PDCD1LG2) in patients with a high and those with a low logistics score. Results indicated that all these immune checkpoints had a higher expression in patients with a high logistics score (**Figures 6A–D**). We also investigated the immune difference in patients with a high and those with a low logistics score. In the combined and IMvigor210 cohorts, we all observed a higher level of M2 macrophages in patients with a high logistics score (**Figures 6E, F**). Next, results showed that M2 macrophages were positively correlated with PTHLH, BHMT2, NGFR, and logistics score (**Figures 6G–J**).

### Patients with a high logistics score might have a higher genomic instability

**Figure 7:**
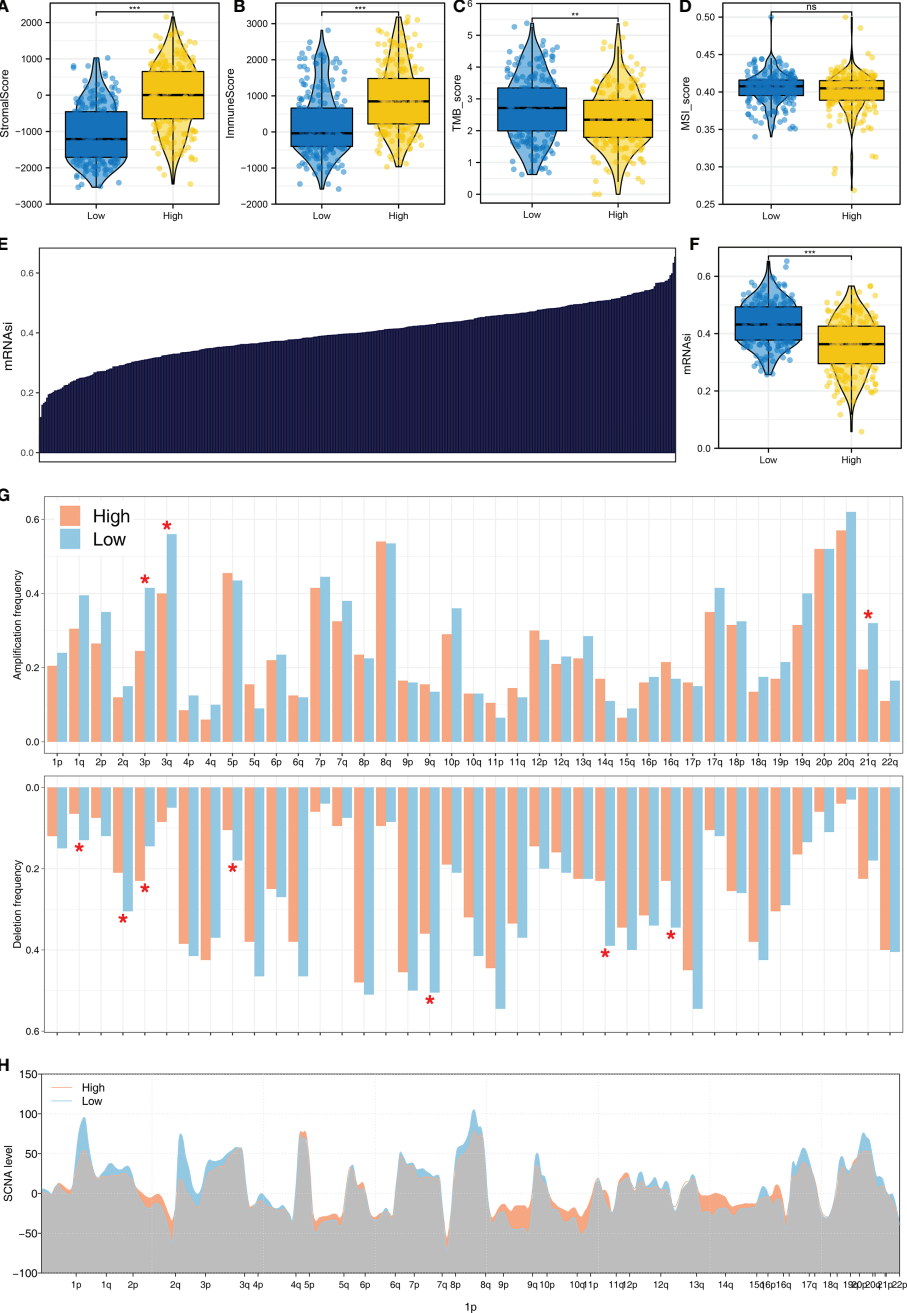
The genomic difference in patients with a high and those with a low logistics score **(A)** Level of stromal score in patients with a high and those with a low logistics score. **(B)** Level of immune score in patients with a high and those with a low logistics score. **(C)** Level of TMB score in patients with a high and those with a low logistics score. **(D)** Level of stromal score in patients with a high and those with a low logistics score. **(E)** The obtained mRNAsi in the TCGA database. **(F)** Level of mRNAsi score in patients with a high and those with a low logistics score. **(G, H)** Comparisons of arm-level amplification and deletion frequencies and focal-level amplification and deletion levels in patients with high and those with low logistics scores. *P < 0.05, **P < 0.01, ***P < 0.001. ns: P > 0.05.

Moreover, we observed a higher stromal score, immune score, TMB score, and MSI score in patients with a high logistics score (**Figures 7A–D**). The mRNAsi obtained is shown in **Figure 7E**. Patients with a high logistics score might have a higher mRNAsi (**Figure 7F**). Meanwhile, we found that the patients with a high logistics score might have a lower level of amplification frequency in the 3p, 3q, and 21q sites in the chromosome (**Figure 7G**). In the deletion frequency, a lower level in the 1q, 2q, 5p, 9q, 14q, and 16q sites were found in patients with a high logistics score (**Figure 7G**). Meanwhile, the difference in SCNA level was also illustrated (**Figure 7H**).

### PTHLH was associated with patients’ survival and M2 macrophage polarization

**Figure 8:**
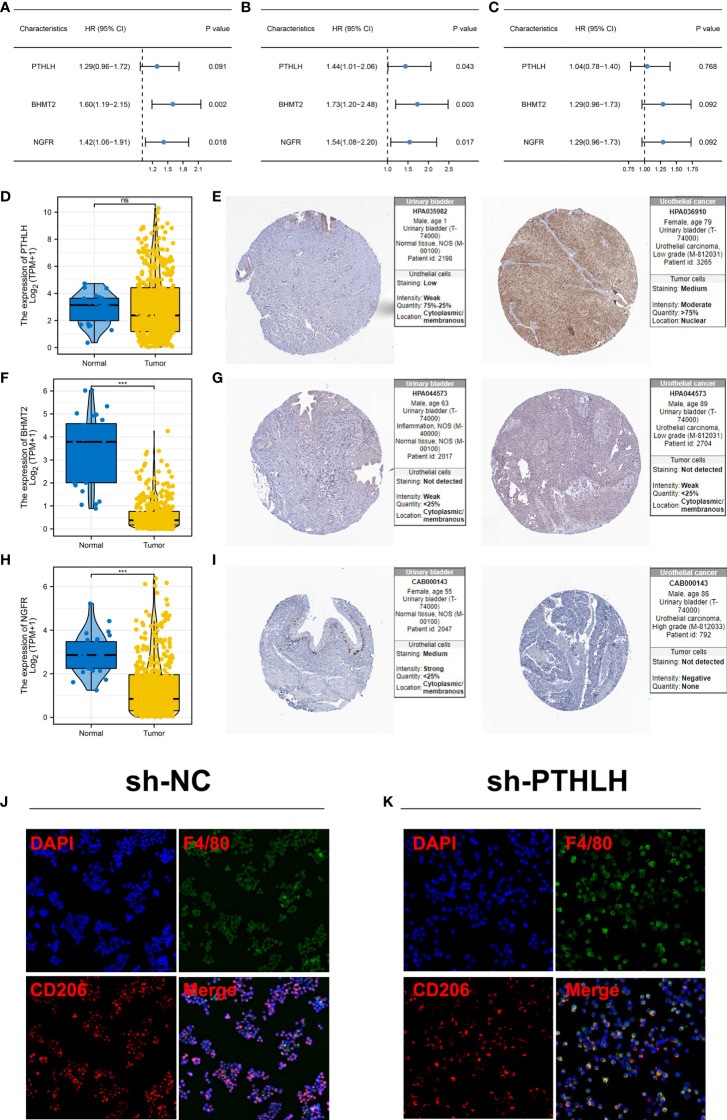
Further exploration of PTHLH, BHMT2, and NGFR **(A)** Prognosis correlation of PTHLH, BHMT2, and NGFR in overall survival. **(B)** Prognosis correlation of PTHLH, BHMT2, and NGFR in disease-free survival. **(C)** Prognosis correlation of PTHLH, BHMT2, and NGFR in progression-free survival. **(D)** Expression level of PTHLH in normal bladder and bladder cancer tissue in mRNA level. **(E)** Representative immunohistochemistry image of PTHLH protein level in normal bladder and bladder cancer tissue. **(F)** Expression level of BHMT2 in normal bladder and bladder cancer tissue in mRNA level. **(G)** Representative immunohistochemistry image of BHMT2 protein level in normal bladder and bladder cancer tissue. **(H)** Expression level of NGFR in normal bladder and bladder cancer tissue in mRNA level.; **(I)** Representative immunohistochemistry image of NGFR protein level in normal bladder and bladder cancer tissue. **(J, K)** Knockdown of PTHLH in bladder cancer cells can significantly inhibit the M2 polarization of co-culture M0 macrophages. ***P < 0.001. ns: P > 0.05.

Following this, we investigated the prognostic role of PTHLH, BHMT2, and NGFR. Results showed that all these three genes were risk factors for overall survival and disease-free survival, but not for progression-free survival (**Figures 8A–C** and **Figure S4**). Then, we try to identify the expression difference of these molecules in bladder cancer tissue. No significant difference was found in PTHLH mRNA level between normal and bladder cancer tissue (**Figure 8D**). However, the HPA database showed a high protein level of PTHLH in bladder cancer tissue (**Figure 8E**). For the mRNA level, BHMT2 was lower in bladder cancer tissue, but not in protein levels (**Figures 8F, G**). Also, the same trend was noticed in NGFR (**Figures 8H, I**). Considering the high protein level of PTHLH in bladder cancer tissue, we next try to validate its association with M2 macrophages. The knockdown efficiency is shown in **Figure S5**. Immunofluorescence showed that knockdown of PTHLH in bladder cancer cells can significantly inhibit the M2 polarization of co-culture M0 macrophages (**Figures 8J, K**).

## Discussion

There is no doubt that bladder cancer represents one of the greatest health problems globally (18). In recent years, immunotherapy, such as immune checkpoint inhibitors, has improved bladder cancer treatment options.

Here, through the combined (TCGA, GSE128959, GSE13507, and GSE83586) and IMvigor210 cohorts, we comprehensively investigated the biological and immune microenvironment differences in patients with diverse immunotherapy responses. Meanwhile, we found that M2 macrophage could affect the sensitivity of bladder cancer patients to immunotherapy. Moreover, PTHLH, BHMT2, and NGFR were identified, which all have good prediction abilities for patients’ immunotherapy. Then, a logistics regression model was established based on PTHLH, BHMT2, and NGFR, and each patient was assigned a logistics score. Afterwards, we explored the difference in patients with a high and those with a low logistics score, including biological enrichment, immune microenvironment, and genomic characteristics. Meanwhile, data from the HPA database indicated a higher protein level of PTHLH in bladder cancer tissue. Immunofluorescence showed that the knockdown of PTHLH in bladder cancer cells can significantly inhibit the M2 polarization of co-culture M0 macrophages.

Based on the immune analysis, we found that M2 macrophages might affect bladder cancer immunotherapy. Tissue homeostasis is mediated by macrophages. Meanwhile, cancer-associated macrophages show the ability to hamper T-cell recruitment and its function, as well as regulate other aspects of tumor immunity (19). Previous studies have reported the potential effects of macrophages in tumor immunotherapy (20). The effect of TAMs on naive T-cell proliferation has been demonstrated in numerous studies, which suggests that macrophages can suppress T-cell function directly (21). An example is that arginase-1 is a common marker of M2 macrophage in mice, which was also involved in antitumor activity and T-cell fitness (22). Meanwhile, in ovarian cancer, Curiel et al. found that macrophages could mediate the recruitment of Treg cells through secreting CCL22, contributing to immunosuppression (23). In breast cancer, macrophages characterized by the expression of IL10 could hamper CD8+ T-cell-dependent responses by inhibiting IL-12 expression in intratumoral dendritic cells (24).

We identified three genes, PTHLH, BHMT2, and NGFR, involved in bladder cancer immunotherapy. Also, the logistics model based on these three genes can effectively indicate immunotherapy sensitivity and patient prognosis. Moreover, we found that all these three genes were positively correlated with M2 macrophage infiltration. In renal cancer, Yao et al. demonstrated that the upregulation of PTHLH can indicate more progressive clinical features and poor prognosis (25). In pancreatic cancer, the protein named PTHrP (encoded by PTHLH) can drive the growth of primary and metastatic tumors in mice (26). In colon cancer, Chen et al. indicated that NGFR could act as a tumor suppressor by activating S100A9, thus leading to the enhanced apoptotic and autophagic effects of 5-fluorouracil (27). Huang et al. found that the NGFR-FOXP3 positive feedback loop contributes to ICOTINIB resistance in non-small cell lung cancer (28). Also, we found that PTHLH can induce M2 macrophage polarization. Our results indicated that PTHLH, BHMT2, and NGFR might be novel targets for bladder cancer immunotherapy and prognosis. In real practice, detecting the relative level of PTHLH, BHMT2, and NGFR through a customized chip or absolute real-time PCR can indicate the sensitivity of immunotherapy and therefore contribute to the therapy option.

Also, we noticed a higher genomic instability in patients with a high logistics score, including TMB, MSI, and tumor stemness index. It is widely believed that TMB is related to the response to immunotherapy for tumors (29). Zhu et al. found that the mutations in EP300 lead to TMB and promote antitumor immunity in bladder cancer (30). Meanwhile, Zhan et al. found that SOX2OT can promote bladder cancer stemness, as well as the malignant phenotype through modulating SOX2 (31). These results indicated that the patients with a high logistics score might have a poor prognosis due to the higher genomic instability.

Even though our research is based on reliable data and analysis, there are some limitations to consider. Firstly, our analysis primarily had a Western population, and therefore, underlying race bias might reduce the credibility of conclusions. Secondly, the loss of probes when combining the TCGA and GSE cohorts might result in information loss.

## Data availability statement

Publicly available datasets were analyzed in this study, the names of the repositories and accession numbers are available within the article/**Supplementary Material**.

## Author contributions

WJ, HX, and DG performed the analysis. WJ and BY performed the experiments. CM designed this work. All authors contributed to the article and approved the submitted version.

## Funding

This work was supported by Key research and development project of science and technology department of Sichuan province (Number 2022YFS0156).

## Conflict of interest

The authors declare that the research was conducted in the absence of any commercial or financial relationships that could be construed as a potential conflict of interest.

## Publisher’s note

All claims expressed in this article are solely those of the authors and do not necessarily represent those of their affiliated organizations, or those of the publisher, the editors and the reviewers. Any product that may be evaluated in this article, or claim that may be made by its manufacturer, is not guaranteed or endorsed by the publisher.

## Supplementary material

Supplementary Figure 1 The flowchart of whole study.

Supplementary Figure 2 KEGG analysis of immunotherapy responders.

Supplementary Figure 3 Machine learning algorithm – LASSO logistics regression.

Supplementary Figure 4 Prognostic role of PTHLH, BHMT2, and NGFR **(A–C)** Kaplan–Meier survival curves of PTHLH, BHMT2, and NGFR in overall survival. **(D–F)** Kaplan–Meier survival curves of PTHLH, BHMT2, and NGFR in disease-free survival. **(G–I)** Kaplan–Meier survival curves of PTHLH, BHMT2, and NGFR in disease-free survival.

Supplementary Figure 5 Knockdown efficiency of PTHLH.
