# Supplementary figures and images for: Identification and validation of novel biomarkers affecting bladder cancer immunotherapy via machine learning and its association with M2 macrophages
Source: Front Immunol. 2022 Nov 9;13:1051063. doi: 10.3389/fimmu.2022.1051063 (PMC9681792; doi:10.3389/fimmu.2022.1051063)

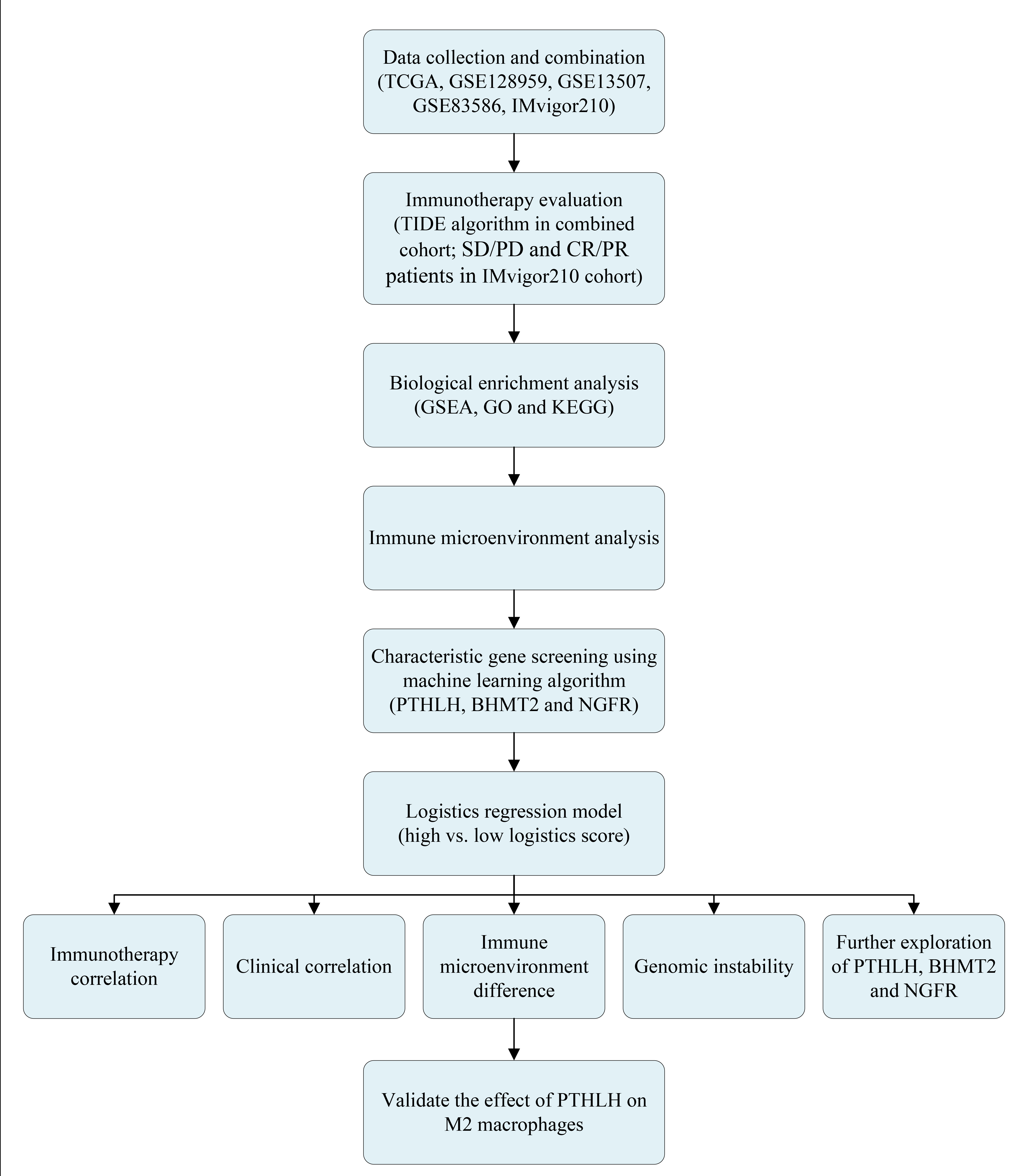

Supplement: Supplementary Figure 1 — The flowchart of whole study. [file Image_1.tif]

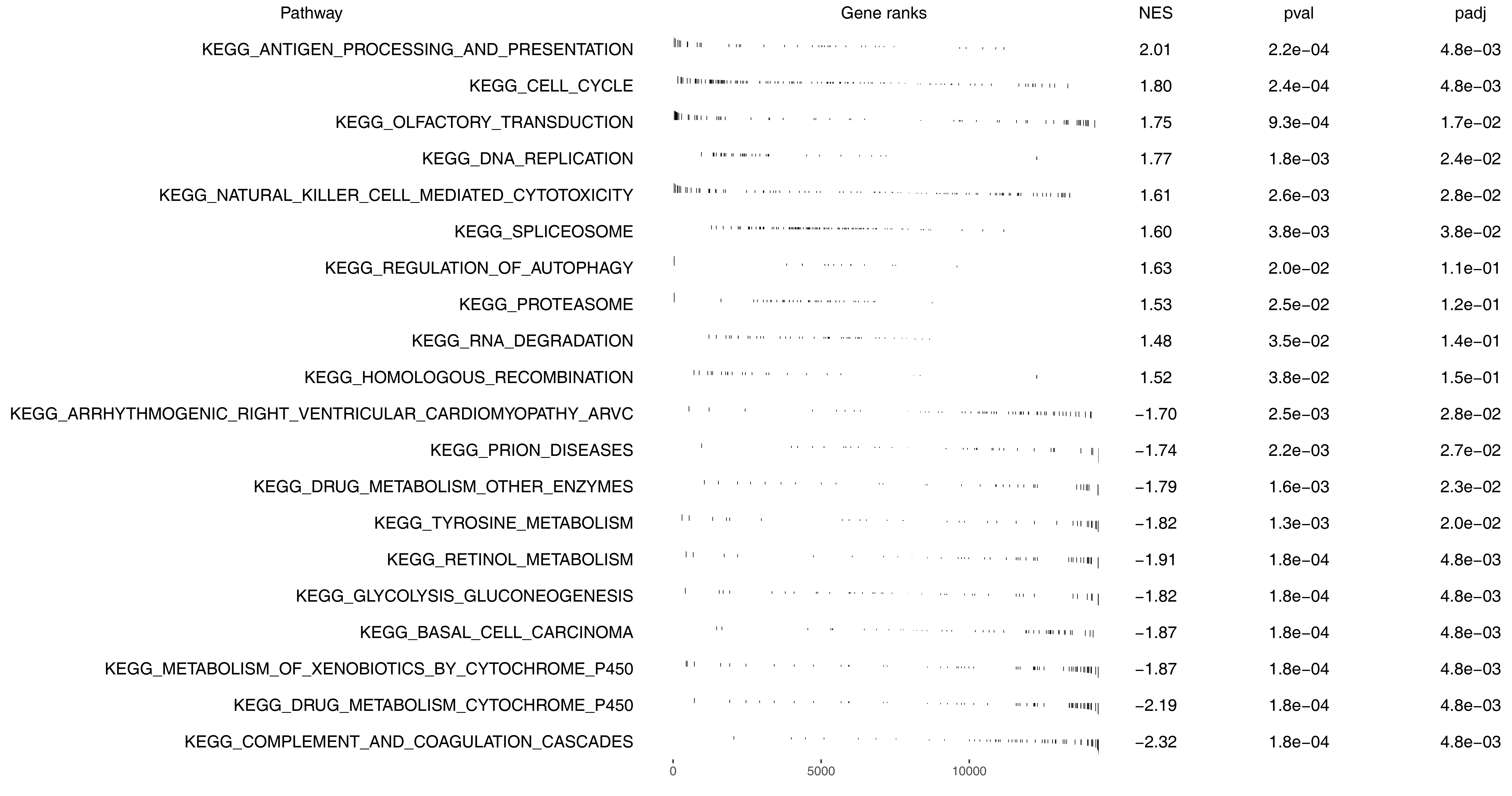

Supplement: Supplementary Figure 2 — KEGG analysis of immunotherapy responders. [file Image_2.tif]

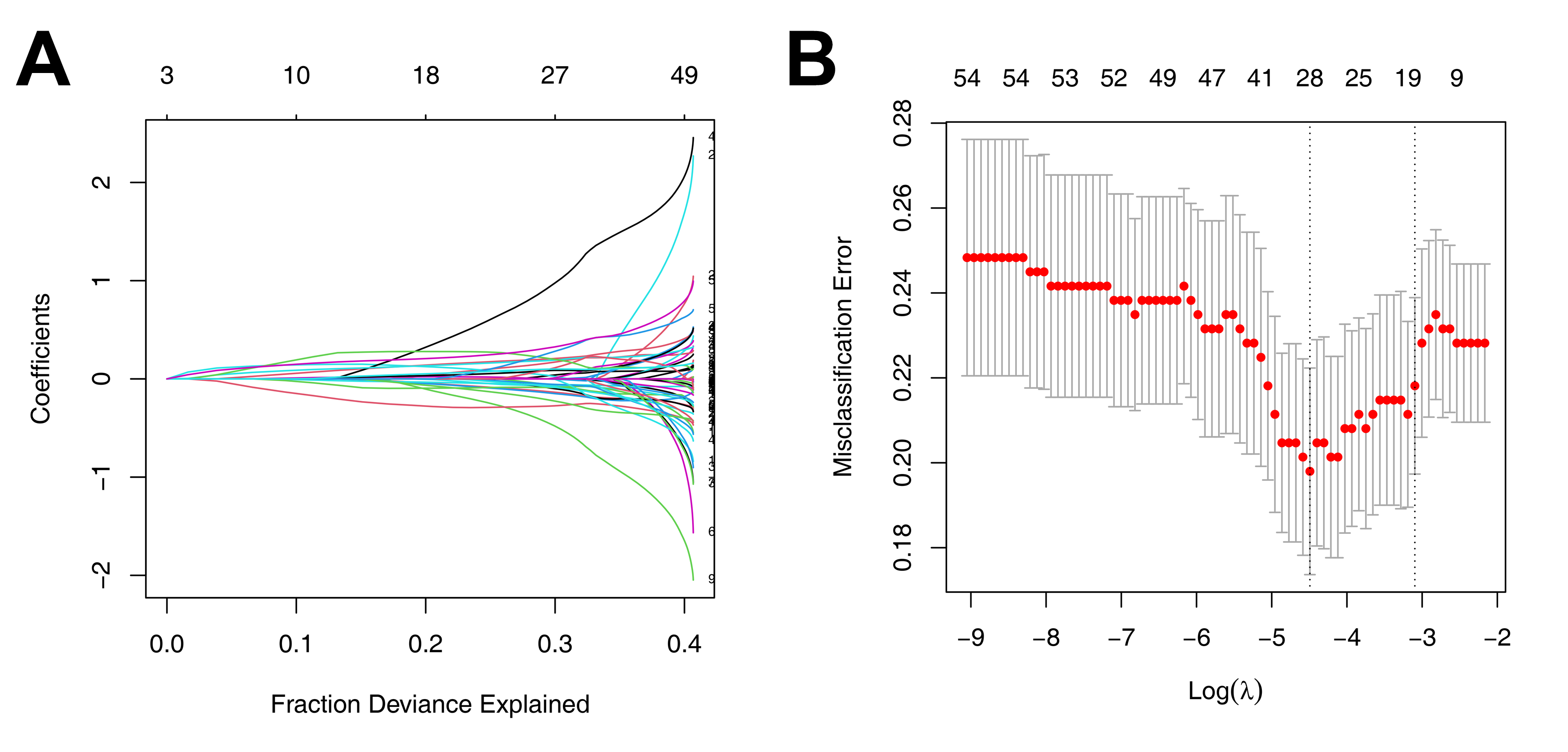

Supplement: Supplementary Figure 3 — Machine learning algorithm – LASSO logistics regression. [file Image_3.tif]

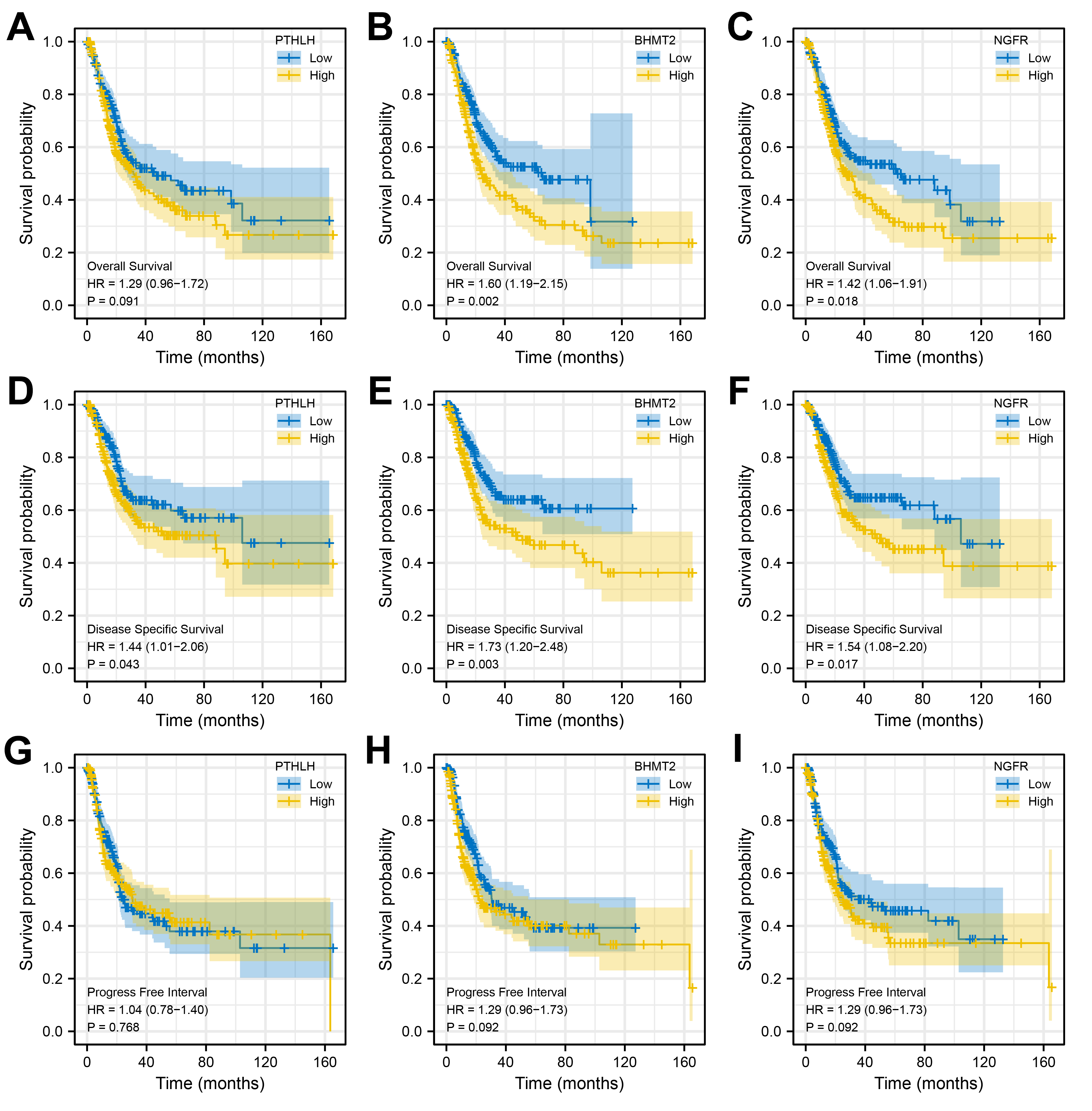

Supplement: Supplementary Figure 4 — Prognostic role of PTHLH, BHMT2, and NGFR (A–C) Kaplan–Meier survival curves of PTHLH, BHMT2, and NGFR in overall survival. (D–F) Kaplan–Meier survival curves of PTHLH, BHMT2, and NGFR in disease-free survival. (G–I) Kaplan–Meier survival curves of PTHLH, BHMT2, and NGFR in disease-free survival. [file Image_4.tif]

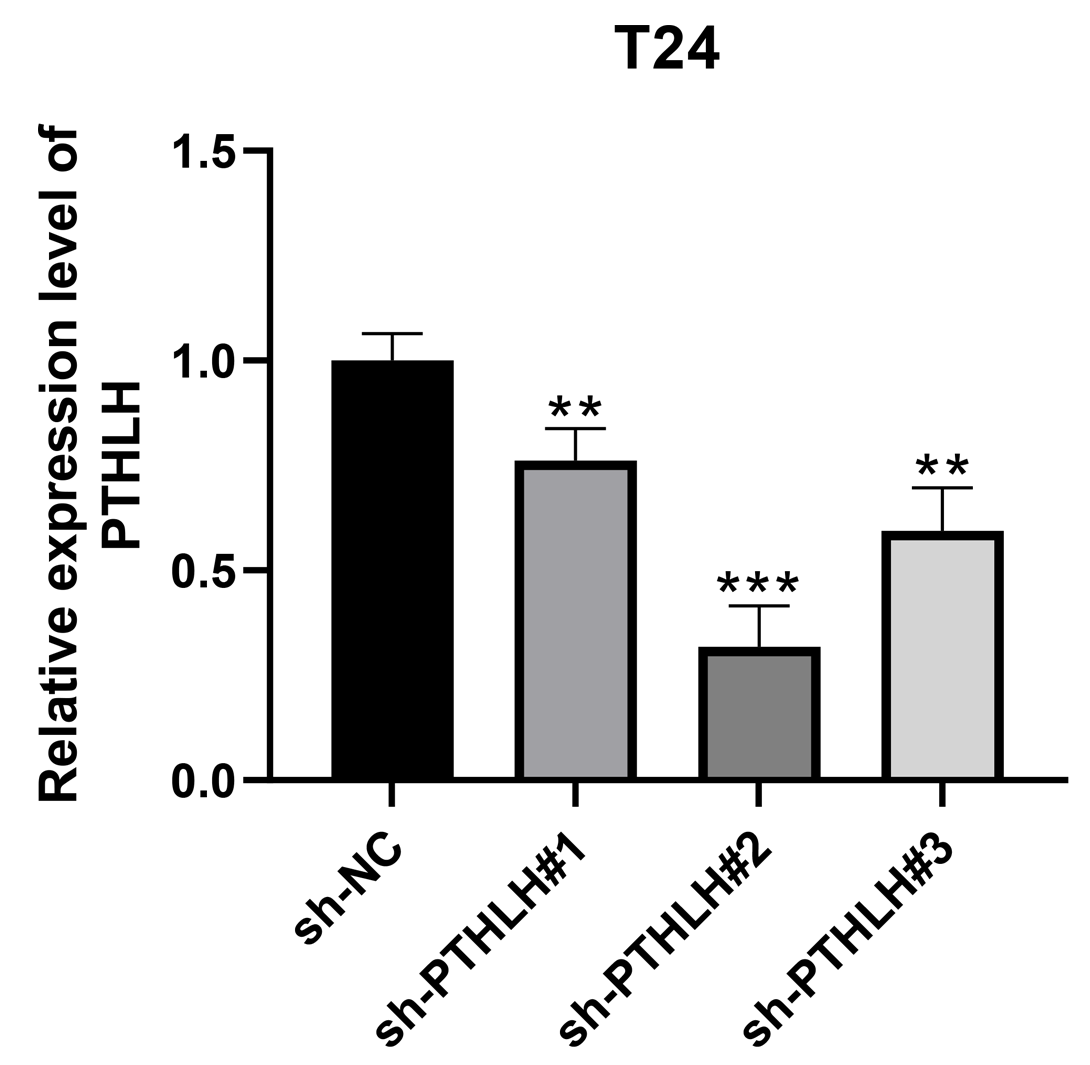

Supplement: Supplementary Figure 5 — Knockdown efficiency of PTHLH. [file Image_5.tif]
